# Cell-free electrophysiology of human VDACs incorporated into nanodiscs: An improved method

**DOI:** 10.1016/j.bpr.2021.100002

**Published:** 2021-07-02

**Authors:** Stefano Conti Nibali, Maria Carmela Di Rosa, Oliver Rauh, Gerhard Thiel, Simona Reina, Vito De Pinto

**Affiliations:** 1 Department of Biomedical and Biotechnological Sciences, University of Catania, Catania, Italy; 2 Membrane Biophysics and Center for Synthetic Biology, Technische Universität Darmstadt, Darmstadt, Germany; 3 Department of Biological, Geological and Environmental Sciences, Section of Molecular Biology, University of Catania, Catania, Italy; 4 we.MitoBiotech.srl, Catania, Italy

## Abstract

Voltage-dependent anion-selective channel (VDAC) is one of the main proteins of the outer mitochondrial membrane of all eukaryotes, where it forms aqueous, voltage-sensitive, and ion-selective channels. Its electrophysiological properties have been thoroughly analyzed with the planar lipid bilayer technique. To date, however, available results are based on isolations of VDACs from tissue or from recombinant VDACs produced in bacterial systems. It is well known that the cytosolic overexpression of highly hydrophobic membrane proteins often results in the formation of inclusion bodies containing insoluble aggregates. Purification of properly folded proteins and restoration of their full biological activity requires several procedures that considerably lengthen experimental times. To overcome these restraints, we propose a one-step reaction that combines in vitro cell-free protein expression with nanodisc technology to obtain human VDAC isoforms directly integrated in a native-like lipid bilayer. Reconstitution assays into artificial membranes confirm the reliability of this new methodological approach and provide results comparable to those of VDACs prepared with traditional protein isolation and reconstitution protocols. The use of membrane-mimicking nanodisc systems represents a breakthrough in VDAC electrophysiology and may be adopted to further structural studies.

## Graphical abstract

**Figure:**
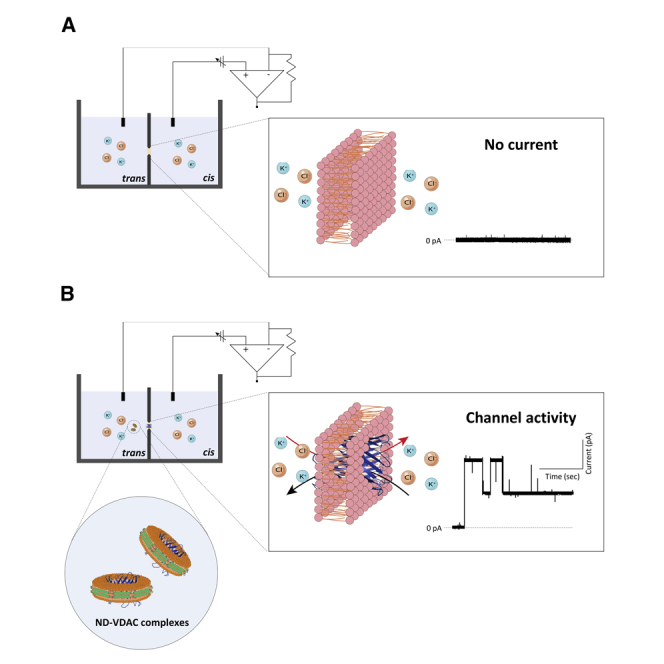

## Why it matters

This work demonstrates the extraordinary advantages in terms of reproducibility and experimental effectiveness in combining in vitro VDAC translation with nanodisc technology. Accordingly, single-channel recordings as well as voltage dependence and selectivity measurements of the three human VDAC isoforms in planar lipid bilayer completely reproduced and even improved results obtained with conventional expression and reconstitution systems. In particular, electrophysiological features of human VDAC3 were in-depth investigated by means of a cysteine-less mutant that validated the extreme importance of cysteine residue in pore functionality, as already reported in literature.

## Introduction

VDACs (voltage-dependent anion-selective channels) are aqueous pore-forming proteins that mediate communication across the outer mitochondrial membrane of all eukaryotes (1). Interactions with cytosolic enzymes (2,3) and both antiapoptotic and proapoptotic factors make VDAC a key protein in regulating mitochondrial metabolism and apoptosis (4, 5, 6, 7). In mammals, evolution led to three isoforms: VDAC1, VDAC2, and VDAC3, encoded by three genes located on different chromosomes (8). Although sharing ∼70% sequence homology, VDAC isoforms fulfill distinctly different physiological roles. VDAC1 is the main isoform responsible for membrane permeability and additionally interacts with Bcl-2 proapoptotic proteins and with hexokinase (9,10); VDAC2 was initially considered an antiapoptotic protein (11) before later contrasting evidence showed that it interacts with Bax, a proapoptotic protein (12). However, there is no doubt that they play a relevant role in the control of cell death. VDAC3 has been proposed to be involved in reactive oxygen species (ROS) homeostasis and mitochondria quality control (13,14). According to the three-dimensional structure, VDAC1 exhibits a transmembrane *β*-barrel architecture composed of 19 amphipathic *β*-strands together with a N-terminal *α*-helix moiety folded inside the pore (15, 16, 17). The *α*-helix is part of the voltage sensor and is essential for channel gating (18). Electrophysiological properties of VDACs have been extensively examined exploiting the planar lipid bilayer (PLB) technique (19, 20, 21). VDACs spontaneously insert into PLBs, where they form pores with an average conductance of ∼4 nS in 1 M KCl. Low membrane potentials (0 ± 20 mV) maintain channels in a full conducting “open state” that features a considerable preference for anions over cations. Potentials exceeding ±30 mV mediate transition to multiple cation-selective “closed states” with a drastic drop in pore conductance (22, 23, 24). VDAC1 and VDAC2 routinely show this prototypic behavior, whereas VDAC3 reconstitution into PLB has been challenging (25). Checchetto and co-workers first, to our knowledge, described human isoform 3 as a low-conducting pore (conductance ∼100 pA) with no voltage-dependence (26). Further studies uncovered the critical role of cysteine residues in VDAC3 modulating channel activity (14,27,28). Data available so far were derived from the successful membrane incorporation of VDACs isolated from tissue mitochondria (29) or from reconstitution of recombinant proteins (14,26,30). For the latter approach, *Escherichia coli* is the host of choice because of its fast growth and cost-effectiveness, albeit heterologous protein folding failure is not uncommon especially for highly hydrophobic membrane proteins that can aggregate into inclusion bodies (31). Cell-free (CF) protein synthesis (CFPS) systems represent a valid and powerful alternative to avoid protein refolding procedures (32,33). They were initially employed for the exclusive production of soluble proteins (34, 35, 36): CFPS systems have subsequently emerged as a suitable tool also for the high-throughput expression of membrane proteins thanks to the development of lipid membrane mimics (e.g., detergent micelles, lipid/detergent mixtures, liposomes, and nanodiscs (NDs)) (37,38). NDs are the most recent class of model membrane systems, structurally composed of a discoidal phospholipid bilayer which is stabilized in solution by two pairs of amphipathic helical membrane scaffold proteins (MSPs) (39). In the last decades, useful application of NDs for CF expression of membrane proteins has been reported. NDs offer a native-like environment for maintaining the structure and functionality of membrane proteins in solution (40), providing a worthy condition for functional analysis in PLBs (41, 42, 43, 44). ND-embedded VDAC1 and VDAC2 were already investigated in (45) and (46) for structural studies by solution NMR and functional assays with PLBs, respectively. In both cases it appeared that the structural and functional properties of the VDACs in NDs were not different from micelle-embedded VDACs. To date, however, reports about the use of the CFPS-NDs binomial technique in VDAC electrophysiology are missing. In this work, we combine for the first time the in vitro protein synthesis system with the ND technology to express and reconstitute the three human VDAC isoforms (VDAC_CF/ND_) into artificial lipid membranes. In addition, we compared the impact of reducing agents in the buffer and the removal of cysteines on the biophysical properties of hVDAC3 from the combined in vitro translation/ND method (hVDAC3_CF/ND_) with those obtained by canonical recombinant production and protein isolation protocols (14,28). Our results clearly indicate that this innovative method keeps the electrophysiological properties of VDAC unchanged, although it reduces experimental times and increases production yield. Despite a vast literature with detailed biophysical analysis of functional properties of VDAC channels, the technical approach proposed here represents a novel, to our knowledge, and promising method for an easier, quicker, and more reliable investigation of VDAC function in PLBs.

## Materials and methods

### CF cloning of hVDAC1, hVDAC2, hVDAC3, and hVDAC3 C0A

The coding sequence of human VDAC1, VDAC2, VDAC3, and VDAC3 C0A obtained from pET21a vector (Novagen) were amplified by PCR and cloned into the pET24Δlac vector (Merck, Darmstadt, Germany) with the NEBuilder HiFi DNA Assembly Master Mix (New England BioLabs). The following pairs of primers were used for cloning (Table 1).

**Table 1:** List of primers used for cloning

Primer	Sequence
Fw hVDAC1	5′-GTTTAACTTTAAGAAGGAGATATACATA TGGCTGTGCCACCCACGT-3′
Rev hVDAC1	5′-CAGCATGGACCACAGCAGTCGACCTATGCT TGAAATTCCAGTCCTA-3′
Fw hVDAC2	5′-GTTTAACTTTAAGAAGGAGATATACATA TGGCGACCCACGGACAGACT-3′
Rev hVDAC2	5′-CAGCATGGACCACAGCAGTCGACCTAAG CCTCCAACTCCAGGGCGA-3′
Fw hVDAC3	5′-GTTTAACTTTAAGAAGGAGATATACATA TGTGTAACACACCAACGT-3′
Rev hVDAC3	5′-CAGCATGGACCACAGCAGTCGACCTAAG CTTCCAGTTCAAATCCCA-3′
Fw hVDAC3 C0A	5′-GTTTAACTTTAAGAAGGAGATATACATAT GGCTAACACACCAACGT-3′
Rev hVDAC3 C0A	5′-CAGCATGGACCACAGCAGTCGACCTAAGC TTCCAGTTCAAATCCCA-3′

### Protein expression and purification

Heterologous expression of recombinant human VDAC1 cloned in pET21a vector was performed as already reported in (14,26,47). The C-terminal His*-*tagged VDAC1 was purified by a single-step affinity chromatography using a Ni-NTA agarose (Qiagen, Hilden, DE) packed column according to the manufacturer’s instructions and then refolded as described in (14,26,47). CF expression of human VDAC1, VDAC2, VDAC3, and VDAC3C0A was achieved using the MembraneMax HN Protein Expression Kit (Invitrogen, Carlsbad, CA) in the presence of NDs with a DMPC (1,2-dimyristoyl-sn-glycero-3-phosphocholine) bilayer. The scaffold proteins of the NDs contained a His-tag for purification. Briefly, 35 *μ*M of MSP2N2-his or MSP1D1-his NDs (Cube Biotech, Monheim, DE) were added to the CFPS mixture and incubated at 37°C for 3.5 h in an orbital shaker at 1000 rpm. VDAC-ND complexes were then purified using Ni-NTA affinity chromatography and every purification step was carried out in the absence of any reductant. After the addition of 400 *μ*L of equilibration buffer (10 mM imidazole, 300 mM KCl, 20 mM NaH_2_PO_4_, pH 7.4) the whole reaction mix was loaded onto a pre-equilibrated 0.2 HisPur Ni-NTA agarose spin column (Thermo Fisher Scientific, Rockford, IL) and incubated for 1 h at room temperature at 200 rpm. Afterwards, the buffer was removed by centrifugation at 700 × g for 2 min and the column was washed three times with 400 *μ*L of washing buffer (20 mM imidazole, 300 mM KCl, 20 mM NaH_2_PO_4_, pH 7.4) to remove unspecific binders. The His-tagged NDs containing VDAC were eluted with 600 *μ*L of elution buffer (250 mM imidazole, 300 mM KCl, 20 mM NaH_2_PO_4_, pH 7.4) and collected in three 200 *μ*L fractions. Purified hVDACs assembled into NDs were diluted in NuPage LDS buffer with reducing agent, heated at 95°C for 5 min and separated onto a 4–12% NuPage Bis-Tris gel (Thermo Fisher Scientific, Carlsbad, CA) in MES running buffer at 200 V. Samples were stored at 4°C for up to 12 days.

### PLB

Electrophysiological analysis of recombinant hVDAC1 was performed as previously described (14,26,47,48). Briefly, an artificial PLB made of 1% DiPhPC (Avanti Polar Lipids, Alabaster, AL) in n-decane was formed on an aperture of 200 *μ*m in a Derlin cuvette (Warner Instruments, Hamden, CT). Membrane capacitances of 110–150 pF were accepted for proper lipid bilayers. Channel insertion was obtained by addition of ∼40 ng of refolded protein solution to the *cis* side of the cuvette containing 3 mL of KCl solution. Data were acquired using a Bilayer Clamp amplifier (Warner Instruments) at 100 *μ*s/point, filtered at 300 Hz and analyzed using the pClamp software (Ver-10; Molecular Devices, San Jose, CA). Electrophysiological analysis of human VDAC_CF/ND_s was performed using the Innovation setup. An artificial PLB was formed on a hole with a diameter of 100 *μ*m in a 25-*μ*m thick Teflon foil separating two Teflon chambers with a volume of 2.5 mL each. First, the rim of the hole was treated with 1 *μ*L of 1% hexadecane in n-hexane and both chambers were filled to the lower edge of the hole with an electrolyte solution. Subsequently, 35 *μ*L of 15 mg/mL phospholipids dissolved in n-pentane were added to each side of the chamber and bilayers were built using a folding technique that consists in elevating the buffer level of each chamber as reported in (49). Both chambers were connected to the amplifier via Ag/AgCl electrodes. All measurements were performed at RT in 1,2-diphytanoyl-sn-glycero-3-phosphocholine (DPhPC; Avanti Polar Lipids) membranes with symmetrical KCl solution (1 M KCl, 10 mM Hepes, pH 7.0). Membrane capacitances of 100–140 pF were accepted for proper lipid bilayers. Reconstitution of VDAC proteins was observed after the addition of ∼5 *μ*L of the purified channel-ND complex directly below the bilayer in the *trans* compartment with a bent 25 *μ*L Hamilton syringe. The currents were acquired with a sampling frequency of 10 kHz after low-pass-filter at 3 kHz and digitized using an EPC 7 Patch Clamp Amplifier and Patchmaster software (HEKA). Channel conductance (G) was calculated from current (I) measurements in the presence of the applied constant voltage (V) of +10 mV, according to the following equation: conductance (G) = current (I)/voltage (V).

### Voltage dependence analysis

VDAC voltage dependence was measured in symmetrical KCl solution (1 M KCl, 10 mM Hepes, pH 7.0) by applying 10 mHz triangular voltage waves of ±50 mV, time 100 s. At least three independent experiments were performed for each protein. Plots of the average VDAC conductance as a function of voltage were obtained by the application of a voltage range of ±50 mV with discrete steps of ±5 mV for 15 s. The relative conductance was calculated as G/G_0_, where G denotes average conductance at a given Vm and G_0_ denotes average conductance values calculated in the presence of the lowest applied potential. Three independent experiments were performed for each protein. Data are shown as the mean ± SEM and graphited using prism 8.0 software (GraphPad Software).

### Ion selectivity measurement

Ion selectivity measurements were performed in 0.1 M/1 M *cis*/*trans* gradient of KCl and permeability ratios of cation K^+^ (P_K+_) over anion Cl^−^ (P_Cl_^−^) was calculated from the reversal potential (V_rev_) using the Goldman-Hodgkin-Katz equation. Channel insertion was initially achieved in symmetrical 1 M KCl. After the insertion of at least one channel, solution in *cis* was changed perfusing ∼10 chamber volumes and 10 mHz triangular voltage wave (±50 mV; time, 100 s) was applied. The channel conductance in 0.1 M/1 M *cis*/*trans* gradient of KCl was calculated from the current measurements when a voltage Vm is applied, using equation: I = G (Vm − V_rev_).

## Results

### Human VDAC_CF/ND_ isoforms insert into membrane with typical channel conductance

**Figure 1:**
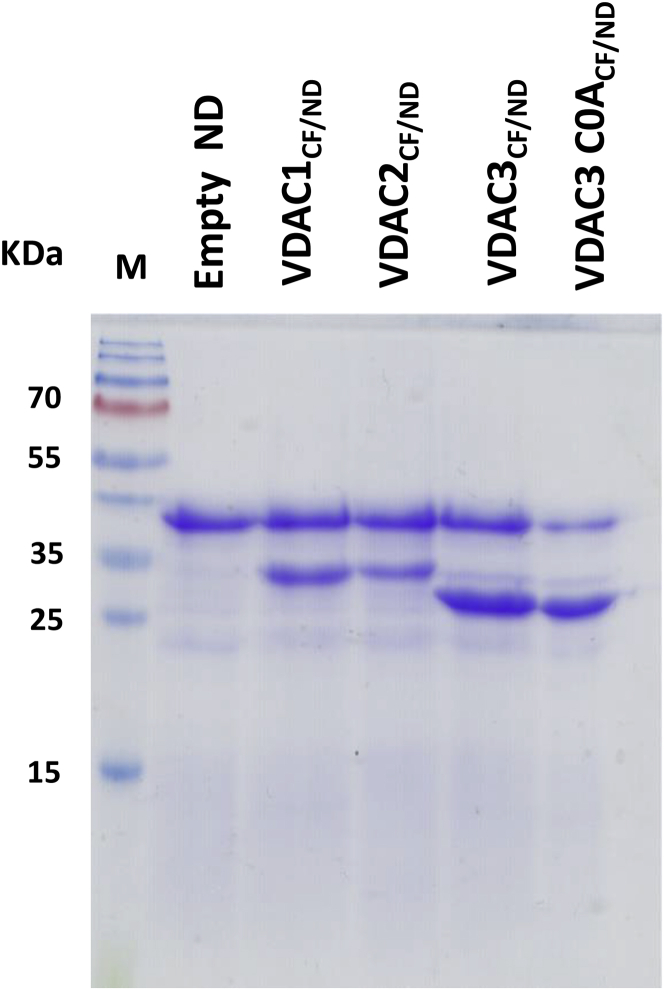
Incorporation of human VDACs into MSP2N2 NDs. 4–12% NuPAGE gel of hVDAC_CF/ND_ isoforms eluted from Ni-NTA columns. The first lane is loaded with 0.5 *μ*L of empty NDs as a control. Lanes 2–5 were loaded with MSP2N2-hVDAC1, -hVDAC2, -hVDAC3, and -hVDAC3 C0A, respectively. In each lane, the protein bands corresponding to the scaffold protein of MSP2N2 NDs (~45 kDa) and those of monomeric human VDACs (~30 kDa) are visible. As expected, VDAC3 wild-type and C0A proteins migrate faster than isoform 1 and 2.

NDs are widely used to mimic a native-like bilayer environment for membrane proteins, thus rendering them soluble in aqueous solutions for structural and functional analysis (50). NDs conceptually arise from high density lipoprotein particles, in particular the apolipoprotein 1 (Apo-1). They consist of a discoidal lipid bilayer which is stabilized and made highly soluble by two pairs of amphipathic membrane scaffold proteins (MSPs), which mimic the function of Apo-1 (51). The length of the MSPs and the stoichiometry of lipid/MSP ratio used in the self-assembly process control the size of the ND structure (39). In recent years, NDs with different lipid composition and size have been developed. In this work, human VDAC proteins were expressed using the CF expression system in the presence of two different commercially available NDs: MSP1D1 and MSP2N2. They share the same dimyristoylphosphatidylcholine (DMPC) bilayer but differ in diameter from ∼9.7 to ∼17 nm, respectively, as measured by solution x-ray scattering (52,53). After the CF expression in the presence of NDs, hVDAC_CF/ND_s were eluted from Ni-NTA columns. The samples were analyzed by NuPAGE (Fig. 1). As shown in Fig. 1, the treatment with the anionic detergent LDS provided two most relevant bands at ∼30 kDa (monomeric hVDACs) and ∼45 kDa (the scaffold protein of MSP2N2 NDs). hVDAC1, hVDAC2, hVDAC3, and hVDAC3 C0A were thus successfully incorporated into MSP2N2 NDs. The experiment was intended to verify the ability of VDAC proteins to incorporate as functional pore into the PLB. The successful electrophysiological recording of typical VDAC channel activity reported below underpins proper assembly and folding of hVDACs produced by the CF protocol in NDs (called from here on hVDAC_CF/ND_). Fig. 1 shows the presence of all three VDAC isoform in the ND preparations, together with the scaffold protein. Hence, the concentration of functional channels in the NDs resulting from this protocol is ideal for reconstituting single VDAC channels into a PLB and for recording its electrophysiological properties. Although the functional reconstitution of human VDACs in MSP2N2 NDs was successful, the same procedure did not generate any channel activity when VDACs were translated in the presence of the smaller MSP1D1 NDs. The absence of channel activity in these experiments can be traced back to the failure of incorporation of VDAC proteins into the smaller MSP1D1 NDs. This is demonstrated by electrophoresis, where in this case exclusively the protein band corresponding to the scaffold protein was detected indicating that these empty NDs did not contain VDACs (data not shown). It is reasonable to speculate that MSP1D1 may form NDs with a diameter too small for hosting a folded VDAC pore.

**Figure 2:**
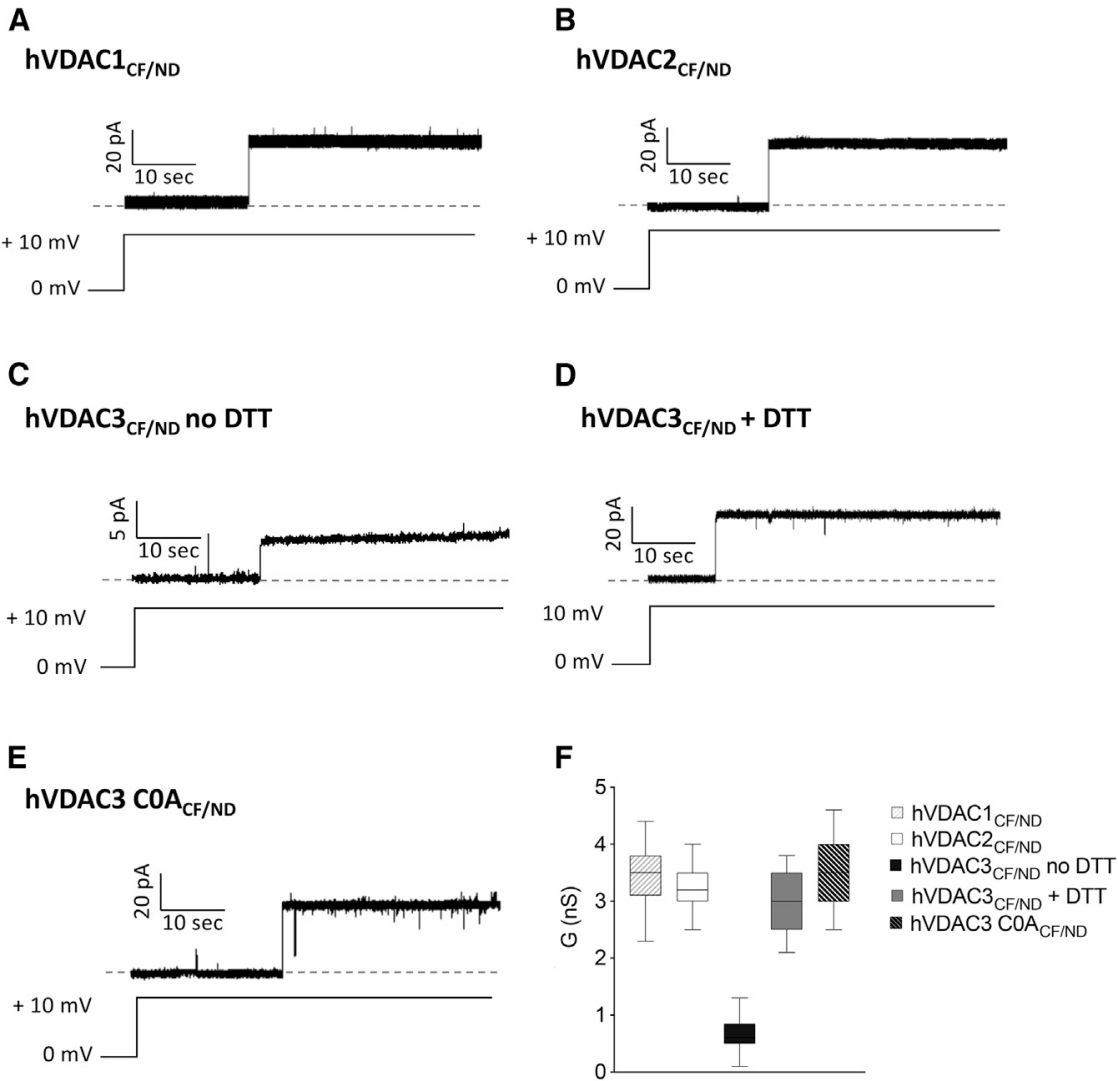
Single-channel recordings of VDAC_CF/ND_. (*A*–*E*) Representative current traces of hVDAC1_CF/ND_ (*A*), hVDAC2_CF/ND_ (*B*), hVDAC3_CF/ND_ no DTT (*C*), hVDAC3_CF/ND_ preincubated with 5 mM DTT (*D*), and hVDAC3 C0A_CF/ND_ (*E*) recorded at +10 mV in 1 M KCl/10 mM HEPES, pH 7.0. (*F*) Distribution of channel conductance events as a function of (G).

Next, we examined the efficiency of the purification protocol and the membrane insertion procedure described above and we tested whether these preparations have the same functional properties of hVDACs prepared by the traditional protocol as in (14,26,47). The VDAC isoforms in NDs were therefore added to the *trans* side of a PLB at a concentration of 100 ng/*μ*L and their channel forming activity analyzed by electrophysiological methods. Channel insertion was studied by applying an electric potential of +10 mV to the membrane. At this voltage, hVDAC1_CF/ND_ and hVDAC2_CF/ND_ inserted as fully open state pores with discrete current steps of 3.47 ± 0.43 nS (*n* = 35) and 3.28 ± 0.36 nS (*n* = 30), respectively (Fig. 2*, A, B, and F*). Fig. 2*, C and F* show that, in the same experimental conditions, hVDAC3_CF/ND_ channels adopted a lower conductance state of 0.64 ± 0.28 nS (*n* = 29), as already noticed in (26). It is worth noting that this state is more frequently observed upon VDAC isoform 3 reconstitution in PLB (14,26,27) and totally differs, as demonstrated below, from the canonical high-conducting state recorded most of the time for VDAC1 and VDAC2 (23,54). Preincubation with 5 mM DTT before bilayer reconstitution significantly increased the mean current through hVDAC3_CF/ND_ (i.e., 3.01 ± 0.47nS (*n* = 30)), albeit the incorporation rate, as empirically observed, was still far below that of isoform 1 and 2 (Fig. 2*, D and F*). Removal of all cysteine residues and their substitution by mutagenesis with alanine (C0A) also causes hVDAC3_CF/ND_ to form channels with the typical VDAC conductance (3.57 nS ± 0.64 (*n* = 33)), and improves the rate of insertion (Fig. 2*, E and F*). These results confirm what was previously reported about the influence of the oxidative state of cysteine residues in VDAC3 on channel conductance (14,26,27).

### NDs preserve the voltage dependence of human VDAC isoforms

VDAC pores show a typical feature, from which their name derives, in that they are voltage dependent. For VDACs this means that, after reconstitution in artificial membranes, the channel conductance does not proportionally raise but begins to decay in response to voltages above 20–30 mV. This phenomenon corresponds to a partial closure of the pore: indeed, such partial “closure” does not allow the passage of large molecules such as ATP and the ion selectivity is modified (24). It is hypothesized that VDAC closure might have relevant functional consequences on the mitochondria activity (1,7,55,56). We aimed to check whether such electrophysiological feature was kept by hVDAC_CF/ND_.

**Figure 3:**
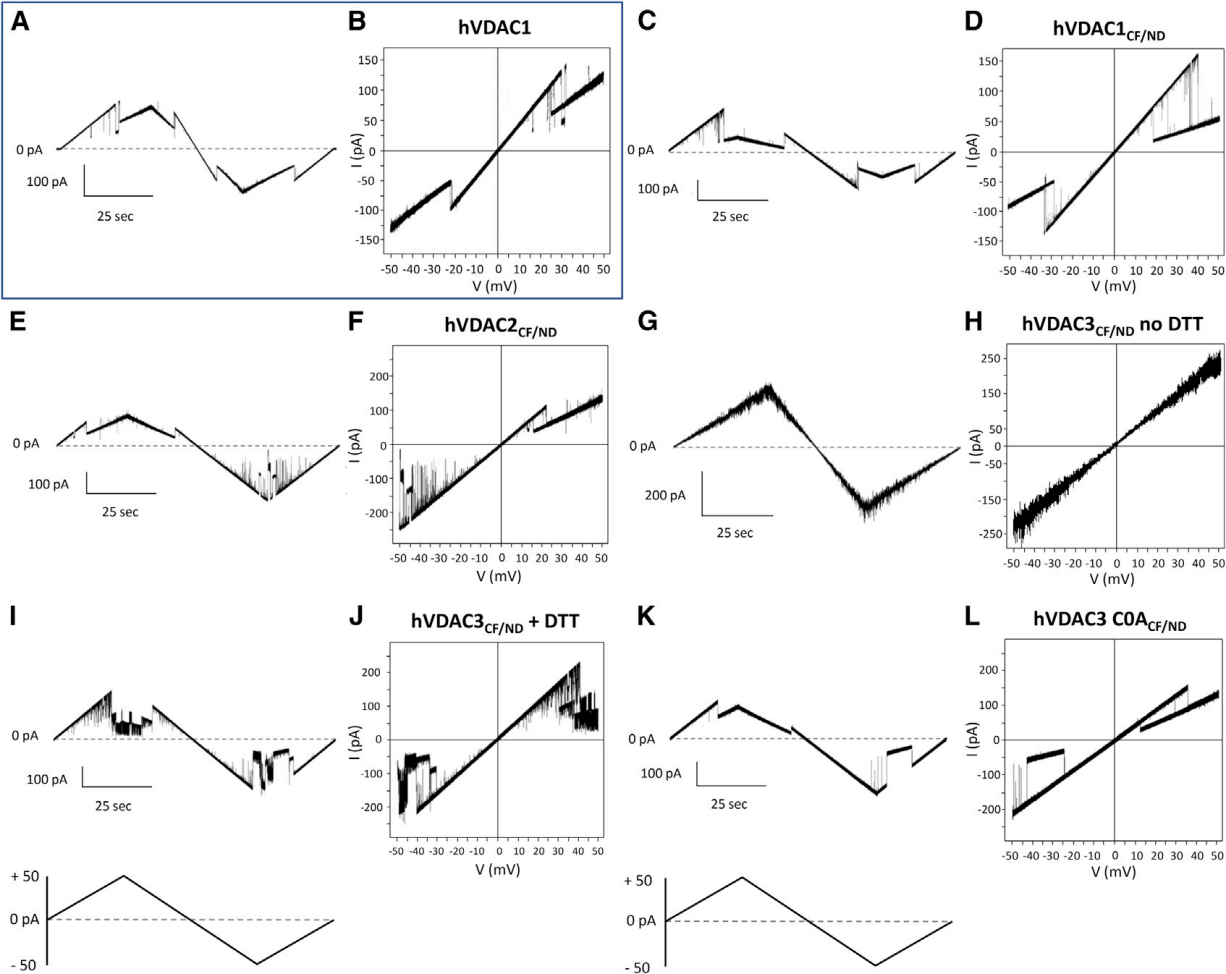
Voltage dependence of human VDAC isoforms analyzed by triangular voltage ramps in PLB in different reconstitution methods. In each experiment, current traces were obtained by applying to the reconstituted VDAC a triangular voltage protocol. The corresponding I–V plots were obtained by plotting the current as a function of clamp voltage. The experiments were performed in symmetrical 1 M KCl solution. (*A* and *B*) hVDAC1: channel refolded and reconstituted as in (14,26,47). (*C* and *D*) hVDAC1_CF/ND_: channel incorporated in NDs as described in Materials and methods. (*E* and *F*) hVDAC2_CF/ND_: conditions as in (*C* and *D*). (*G* and *H*) hVDAC3_CF/ND_ no DTT: multichannel analysis in the absence of any reducing agent; other conditions as in (*C* and *D*). (*I* and *J*) hVDAC3_CF/ND_ + DTT: channel analyzed in the presence of 5 mM DTT; other conditions as in (*C* and *D*). (*K* and *L*) hVDAC3 C0A_CF/ND_: mutagenized hVDAC3 lacking any cysteine, mutated in alanine; other conditions as in (*C* and *D*).

After successful incorporation of VDAC channels from NDs into a host bilayer, we monitored their voltage dependence by applying a triangular voltage ramp from 0 to ±50 mV in 100 s (Fig. 3). At low membrane potentials, hVDAC1_CF/ND_ and hVDAC2_CF/ND_ current traces increased linearly with voltage. Higher voltages of either polarity induced step-like transitions to a single and stable closed substate in hVDAC1_CF/ND_. hVDAC2_CF/ND_ was more responsive to positive voltages than to negative ones: during the short time of the voltage ramp, the channel rapidly shifted to a stable lower conductance substate already at +20 mV. At negative potentials, the channel exhibited first some fast fluctuations at voltages more negative than −30 mV before reaching a steady closed substate between −40 and −50 mV). As found with protein refolded in the traditional way (26), hVDAC3_CF/ND_ did not display any voltage dependence in the absence of reductants. In Fig. 3 *G*, it can be appreciated how current constantly increases and decreases as a function of the driving force (voltage) without showing any gating events. The addition of DTT, as well as the removal of all cysteine residues, made hVDAC3_CF/ND_ sensitive to the membrane potential. As shown in Fig. 3*, I and K*, the application of voltages higher than ±30 mV elicited closures of hVDAC3_CF/ND_ in the presence of 5 mM DTT. The same was observed in the Cys-free hVDAC3 C0A_CF/ND_.

**Figure 4:**
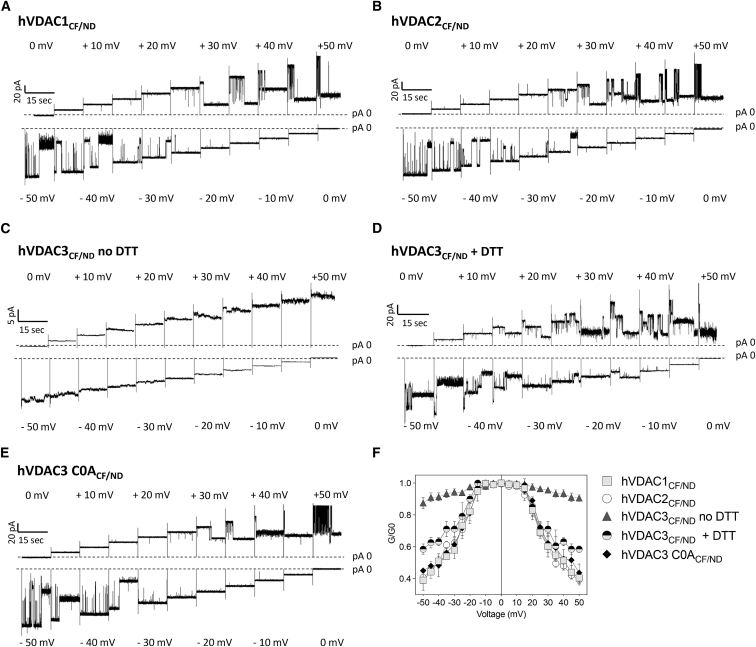
Current traces of reconstituted VDAC isoforms. (*A*–*E*) Current traces of reconstituted hVDAC_CF/ND_ isoforms described as in Fig. 3 were obtained by clamping membrane 15 s voltage steps of ±5 mV over voltage window of ±50 mV. (*F*) Conductance G/G_0_ of VDAC isoforms as a function of the applied voltage. The voltage dependence of most isoforms exhibits a bell shape; only hVDAC3_CF/ND_ shows, in the absence of reducing agents, almost no voltage dependence.

These results, obtained by reconstituting recombinant hVDAC3 from membrane mimetic NDs, support the essential role of cysteines in channel gating, as was previously shown by conventional protocols. Within the applied voltage range +20/–30 mV and +20/–40 mV, hVDAC1_CF/ND_, and hVDAC2_CF/ND_, respectively, are fully open; the linear trajectory of the current follows the voltage ramp. As expected for symmetrical buffers, both in the *cis* and *trans* side the current reverses at 0 mV. At voltages positive and negative outside these windows, partial channel closures can be observed. The voltage-dependent reduction in channel open probability reduces the slope of the I/V curve which translates into a decreased conductance; the decrease in conductance becomes evident at voltages ±20 to 30 mV for hVDAC1_CF/ND_ (Fig. 3 *D*). hVDAC2_CF/ND_ switched to a stable lower conductance substate at positive potentials (+25 mV), whereas at negative potentials, ranging from −30 to −40 mV, the channel exhibited fast open-closed transitions before going into a permanent partially closed substate at voltages higher than −40 mV (Fig. 3 *F*). The I/V plot of hVDAC3_CF/ND_ shows that the channels do not display dependence on the entire voltage range tested because no conductance decrease was recorded. (Fig. 3 *H*). Channel closures at positive and negative voltages are only observed after preincubating hVDAC3 with DTT or after removing its cysteines from the sequence. Under these conditions hVDAC3_CF/ND_ exhibits distinct partial closures at extreme positive and negative voltages. Such closures are evident applying more than ±30 mV to incorporated hVDAC3_CF/ND_ + 5 mM DTT and more than ±35 to 40 mV to hVDAC3 C0A_CF/ND_, respectively (Fig. 3*, J and L*). All data were compared with an experiment in which the triangular voltage wave was applied to hVDAC1 reconstituted by canonical recombinant production and purification from *E. coli* as in (14,26,47) (Fig. 3*, A and B*). The experimental traces of human VDAC isoforms conductance in response to 15 s voltage steps of ±10 mV over a range of ±50 mV, are also shown (Fig. 4). When the normalized conductance G/G_0_ is plotted as a function of V_m_, hVDAC1_CF/ND_, and hVDAC2_CF/ND_ exhibit the characteristic bell-shaped curve. This result indicates a symmetrical voltage-dependent channel closure at both positive and negative potentials (Fig. 4*, A, B, and F*). In the same plot the G/G_0_ ratio of hVDAC3_CF/ND_ remains nearly constant over the entire voltage window (Fig. 4*, C and F*). When the latter channel was incubated under reducing conditions (+DTT) before reconstitution in the bilayer, also hVDAC3_CF/ND_ exhibited a similar bell-shaped voltage dependence as the two other isoforms hVDAC1 _CF/ND_ and hVDAC2 _CF/ND_; even though the voltage dependence of hVDAC3 _CF/ND_ was less steep than for the other isoforms (Fig. 4*, D and F*). An almost perfect overlap of the voltage dependence between all three isoforms was obtained only when the cysteines were removed from the hVDAC3 sequence (hVDAC3 C0A_CF/ND_) (Fig. 4*, E and F*).

### Ion selectivity of VDAC isoforms reconstituted in NDs

**Figure 5:**
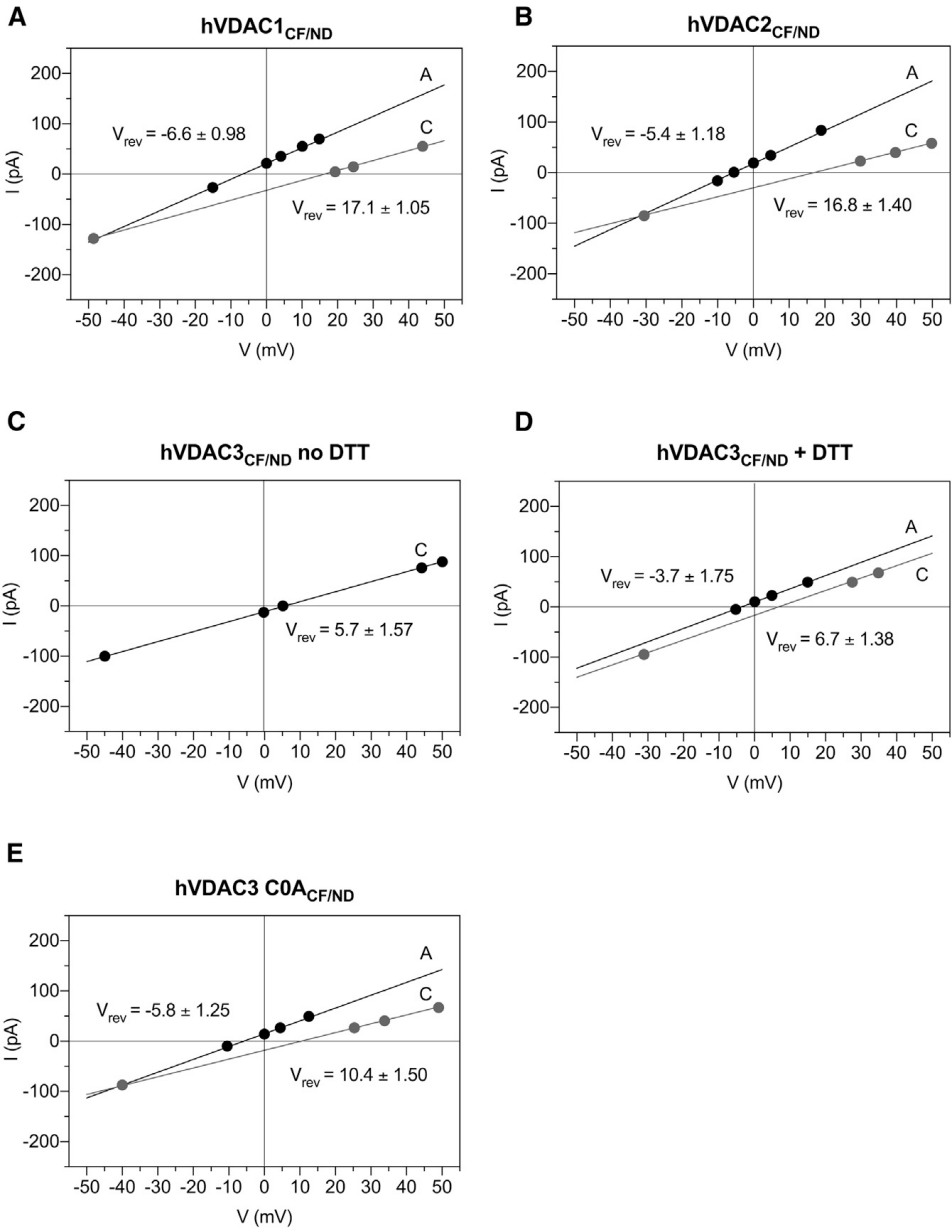
Ion selectivity of hVDAC_CF/ND_s. (*A*–*E*) Representative I/V plots of human VDAC_CF/ND_s performed in a 10-fold gradient of 0.1/1 M KCl. Currents were obtained by application of triangular voltage wave (amplitude ±50 mV). The two different regression lines (*solid lines*), correspond to the canonical anion high-conducting state (A, *black*) and to the cationic low-conducting state (C, *gray*). The intercepts of A and C lines with the I/axis identify the V_rev_-values.

A typical feature of VDACs is that they have a slight preference for anions over cations in the so-called “open state” (20,21) but a cations over anions preference in the “closed state” (24,25). Here, the ion selectivity of the three human VDAC isoforms has been investigated using a 10-fold KCl gradient, as described in Materials and methods. When *cis* and *trans* sides of the membrane contain buffers with different concentrations, the I/V curve of a “selective” channel reverses at a voltage different from zero. The “reversal potential” (V_rev_) at which the current changes its direction is diagnostic for the ion species that carries the current. The I/V plots of hVDAC_CF/ND_ shown in Fig. 5*, A–E* clearly indicate negative V_rev_-values, which underpin the anion selectivity of the “open state.” The “closed state,” in contrast, reveals positive V_rev_-values corresponding to cation selectivity. The permeability ratio of Cl^−^ to K^+^ (P_Cl_^−^/P_K+_) in the “open” and “closed” conformations of human VDAC isoforms was determined from the reversal potential using the Goldman-Hodgkin-Katz equation. In agreement with the literature (20,21,23,24), hVDAC1_CF/ND_ featured a mild anion selectivity in the open state (P_Cl_^−^/P_K+_ = 1.39 ± 0.06, V_rev_ = −6.60 ± 0.98 mV). This preference for anions is reversed to a preferred movement of K^+^ over Cl^−^ (P_Cl_^−^/P_K+_ = 0.45 ± 0.02, V_rev_ = 17.10 ± 1.05 mV) when the channel adopted the lower conducting conformation (“closed state”) (Fig. 5 *A*; Table 2). For hVDAC2_CF/ND_, the calculated V_rev_-values were −5.4 ± 1.18 mV in the open state, corresponding to a permeability ratio P_Cl_^−^/P_K+_ = 1.31 ± 0.08, and 16.8 ± 1.40 mV in the closed state, which is equivalent to a P_Cl_^−^/P_K+_ ratio of 0.43 ± 0.03 (Fig. 5 *B*; Table 2). hVDAC3 deserves a separate discussion.

**Table 2:** Permeability ratio of hVDAC_CF/ND_ complexes

	*P*_*Cl*_^*−*^*/P*_*k+*_*(A)*	*P*_*Cl*_^*−*^*/P*_*k+*_*(C)*
hVDAC1_CF/ND_	1.39 ± 0.06	0.45 ± 0.02
hVDAC2_CF/ND_	1.31 ± 0.08	0.43 ± 0.03
hVDAC3_CF/ND_ no DTT	—	0.76 ± 0.06
hVDAC3_CF/ND_ + DTT	1.24 ± 0.12	0.66 ± 0.06
hVDAC3 C0A_CF/ND_	1.27 ± 0.13	0.64 ± 0.10

The permeability ratio (P_Cl_^−^/P_K+_) of hVDAC_CF/ND_ complexes calculated from corresponding V_rev_ using the Goldman-Hodgkin-Katz equation. Data are means of at least three independent experiments ± SD.

As demonstrated in the previous paragraph, untreated human hVDAC3_CF/ND_ is voltage-insensitive, which provides the possibility to determine the reversal potential exclusively for the conformation in which it inserted. In Fig. 5 *C*, it is indicated that the channel has a V_rev_-value of 5.7 ± 1.57 mV. This corresponds to a permeability ratio P_Cl_^−^/P_K+_ of 0.76 ± 0.06 indicating cation selectivity (Table 2). This finding, however, is not surprising: it is conceivable that a channel with a very small conductance (∼0.64 nS) promotes the passage of K^+^ over Cl^−^ also because of steric hindrance. Preincubation with DTT, which confers the large conductance state, then makes the fully open channel anion-selective with a P_Cl_^−^/P_K+_ = 1.24 ± 0.12 (V_rev_ = −3.7 ± 1.75 mV). The low conductance state reverses in this condition at V_rev_ = 6.7 ± 1.38 mV translating into a cation selectivity of P_Cl_^−^/P_K+_ = 0.66 ± 0.06 (Fig. 5 *D*; Table 2). The fact that the large conductance promotes anion selectivity was further supported by selectivity measurements of hVDAC3C0A_CF/ND_. The reversal potential values of this mutant (V_rev_ = −5.8 ± 1.25 mV) are in the fully open state more similar to hVDAC1_CF/ND_ and hVDAC2_CF/ND_, than to hVDAC3_CF/ND_ no DTT. This reversal voltage translates into a permeability ratio P_Cl_^−^/P_K+_ = 1.27 ± 0.13 in the large-conducting open conformation. The lower conducting closed state of this mutant exhibits a reversal voltage of V_rev_ = 10.4 ± 1.50 mV indicating a cation selectivity with P_Cl_^−^/P_K+_ ratio of 0.64 ± 0.10 (Fig. 5 *E*; Table 2).

## Discussion

**Figure 6:**
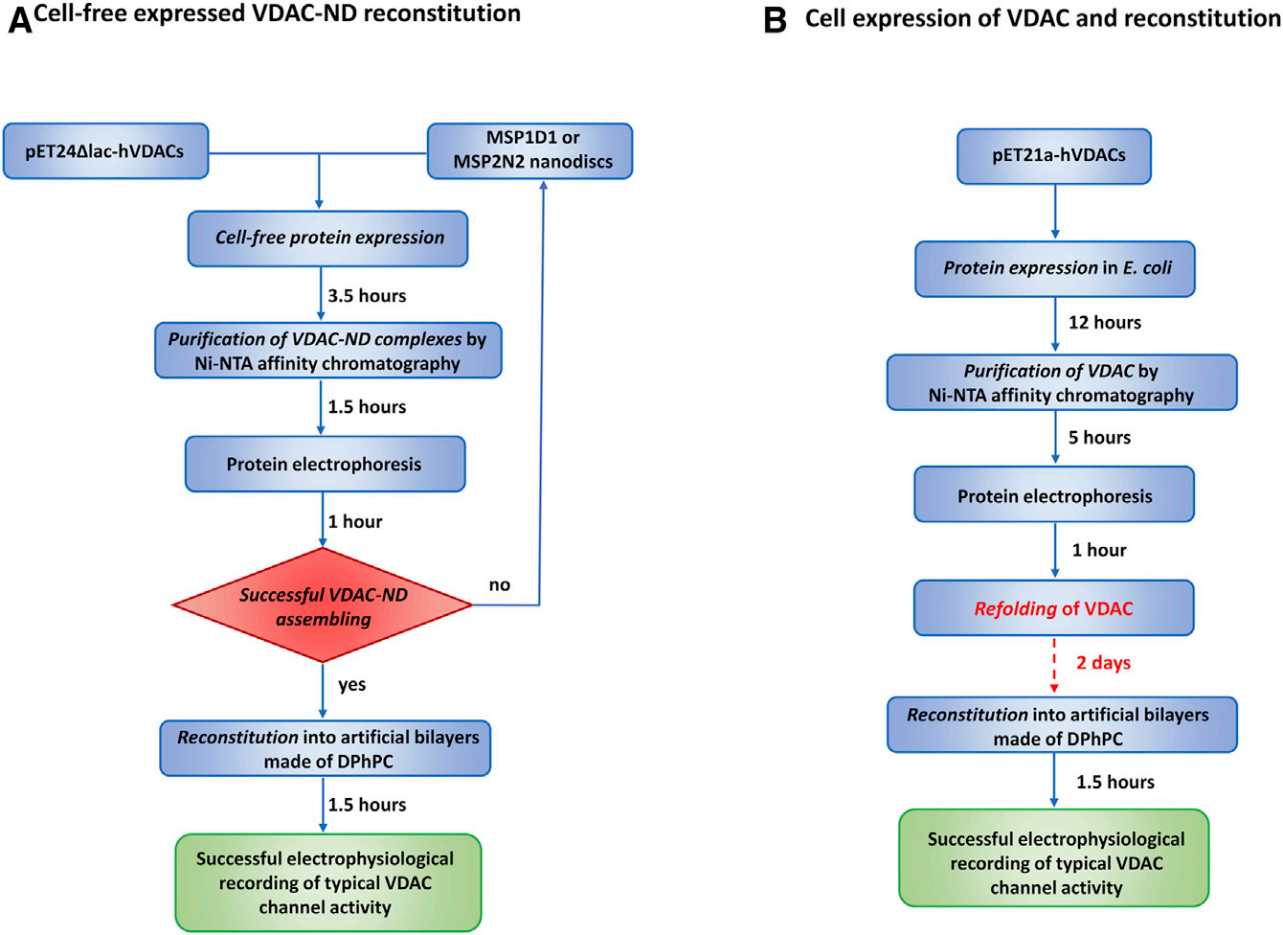
Flowcharts comparing the traditional method consisting of recombinant VDAC expression in microbial systems (right) and the new protocol that combines in vitro translation with nanodisc reconstitution (left). The lack of any type of refolding procedure is responsible for the substancial time gain experienced with the cell-free protein synthesis system.

The first attempt to reconstitute VDAC into a PLB dates back to 1976 (19), when Schein and co-workers accidentally discovered a voltage-dependent and anion-selective channel in the mitochondria extract of *Paramecium Aurelia*. Since then, VDAC electrophysiology has been thoroughly investigated exploiting PLB measures. Today, well-established protocols exist for VDAC incorporation into artificial membranes and all of them involve the use of proteins isolated from tissue mitochondria (20,21,57) or protein produced by microbial systems (26,47). *Escherichia coli* is undoubtedly the most widely used expression platform for the highly efficient production of heterologous proteins. Despite its wide utilization, this technology has limitations that mainly concern the intracellular accumulation of improperly folded proteins in insoluble inclusion bodies and, possibly, the lack of physiological posttranslational modifications. Because functional and structural studies, however, need bioactive proteins, numerous strategies were established to counteract any denaturation process. In the specific case of VDAC, these methods include on-column refolding (30) or drop-wise dilution (14,16,26) in the presence of detergents, along with gel filtration, ion exchange, and size exclusion chromatography (17). Overall, the reported procedures circumvent the difficulties associated with the transmembrane nature of VDAC though, at the same time, substantially prolonging the protein preparation workflow. Furthermore, the presence of contaminants, i.e., channel proteins of the bacterial host, is not absolutely abolished. In the last decades, CFPS has proven to be an excellent platform for in vitro protein expression that avoid all complications associated with living cells. Here, we report, for the first time, CF production of human VDACs and their insertion into NDs. Subsequent reconstitution into PLBs confirms that this procedure maintains the channel function of the proteins. Unlike detergent micelles and liposomes, NDs provide a native-like membrane environment that overcomes heterogeneity and aggregation (58,59). The ND also provides a greater stability for the membrane protein compared with liposomes (60). Furthermore, this technology paves the way for a more accurate control of the local lipid compositions around the integral protein, that, besides, results more active (61). The literature already contains some examples of ND-stabilized VDACs (in particular, VDAC1 (45) and VDAC2 (46)), but all of them still involved the use of conventional bacterial protein expression systems. On the contrary, the results presented in this manuscript indicate the extraordinary advantages in terms of experimental effectiveness in combining in vitro VDAC translation with ND technology. To give a practical example, in vivo VDAC expression systems often require from 4 to 5 days to obtain active recombinant proteins, whereas CF/ND technology extremely cuts down working time to few hours (Fig. 6). The electrophysiological data confirm that human VDAC1_CF/ND_, VDAC2_CF/ND_, and VDAC3_CF/ND_ form pores with robust channel properties. In this regard, VDAC_CF/ND_s registered a higher probability to insert into artificial membranes when compared with VDAC proteins expressed via heterologous system (∼90–70%). The functional features of these channels are undistinguishable from those obtained from recombinant proteins analyzed by canonical PLB protocols. We also checked the influence of PLB lipid composition on the pore-forming activity, by reconstituting hVDAC1_CF/ND_ in a membrane made of 1:1 DiphPG/DiphPC as in (62). There was no difference between the protein produced with the new protocol presented here and the result reported in (62) (data not shown). Accordingly, at low membrane potentials hVDAC1_CF/ND_ and hVDAC2_CF/ND_ easily inserted into Diph-PC membranes as fully “open” channels with average conductance values between 3.47 ± 0.43 nS (*n* = 35) and 3.28 ± 0.36 nS (*n* = 30), respectively. An analysis of the voltage-dependence demonstrated that membrane potentials higher than ±30 mV switched both isoform 1 and 2 to the low-conducting “closed” conformation. Also, the analysis of ion selectivity of channels obtained from the combined CF synthesis/ND system resembled data obtained with conventional methods (63). In both experimental systems the channels provide a weak preference for anion over cations in the high-conducting state that shifts to a distinct cation selectivity in the low conductance state. As expected, human hVDAC3_CF/ND_ exhibited the same peculiar electrophysiological features previously reported for this channel (14,26,28). In the absence of any reductants, the average conductance of reconstituted channels was much lower than those of hVDAC1_CF/ND_ and VDAC2_CF/ND_. Furthermore, the open probability of hVDAC3_CF/ND_ was insensitive to voltage over a wide voltage range. This condition allowed measurements of ion selectivity in a unique channel conformation that emerged as cation-selective. Addition of DTT or replacement of cysteines by alanine (hVDAC3 C0A mutant) converted the human VDAC3_CF/ND_ into the same functional mode of the other two isoforms: the mean current across the hVDAC3_CF/ND_ channel considerably increased both in DTT-treated (3.01 ± 0.47 nS (*n* = 30)) and Cys-less protein (3.57 nS ± 0.64 (*n* = 33)) although with different incorporation rates. Under the aforementioned experimental conditions, hVDAC3_CF/ND_ acquired a voltage-dependence and anion selectivity in the open conformation similar to that of the two other isoforms. The results of these experiments further corroborate the importance of cysteine redox state in pore function (14,26) and they foster the hypothesis that the selectivity of the channel is correlated to the size of the unitary conductance. On the other hand, these experiments show that the new protocol for expression and reconstitution of VDAC pores can perfectly replicates the “classical” one also in altered conditions. In conclusion, we presented here a new protocol of expression and reconstitution of VDAC pores, based on CF expression of the protein and incorporation into NDs, that has the following advantages over the previous methods: 1) it is quicker because it takes only few hours from the expression to the reconstitution in PLB. This is mainly due to the lack of a dedicated refolding procedure, which is quite long and complex for bacterially expressed proteins; 2) consequently it is cheaper; 3) it is easier because the expression and NDs incorporation do not need any complex step in the laboratory; 4) the most interesting future perspective is that a combination of CFPS and reconstitution of active VDACs in NDs paves the way for site-specific incorporation of noncanonical amino acids. This will be interesting for studying structure/function correlations in these channel proteins and for understanding the effects of specific labeling (64). Finally, VDAC forming pores are more stable than those obtained with previous protocols, lasting many days at 4°C without loss of activity or precipitation and are more active in terms of ease of insertion in the PLB and incorporation of undamaged pore-forming protein. All these factors make our protocol an even better strategy than the liposome-fusion proposed by Liguori et al. (65). These authors set up a method for CF production of VDAC within liposomes that, however, requires much longer preparation times because of the need of preventing aggregation of the integral proteins.

The flowcharts in Fig. 6 summarize step by step protocols for the combined in vitro translation and ND reconstitution of VDACs and the conventional approach comprising recombinant VDAC synthesis and purification with advantages and disadvantages of both techniques. In the light of these findings, and of the successful confirmation of full activity recovery showed by the three VDAC isoforms in PLB, which overlaps and even improves recordings of the functional properties reported in conventional system, we believe that our new protocol, combining CF synthesis with NDs reconstitution, will open up new opportunities in VDAC electrophysiology.

## Author contributions

S.C.N. and O.R. performed the electrophysiological experiments on VDAC_CF_ and analyzed the data. M.C.D.R. produced recombinant proteins. G.T., V.D.P., and S.R. designed the experiments and wrote the manuscript.
